# Relationship Between Serum Vitamin D Levels and Chronic Musculoskeletal Pain in Adults: A Systematic Review

**DOI:** 10.3390/nu16234061

**Published:** 2024-11-26

**Authors:** José Luís Alonso-Pérez, Iker Martínez-Pérez, Carlos Romero-Morales, Vanesa Abuín-Porras, Ruben López-Bueno, Giacomo Rossettini, Massimiliano Leigheb, Jorge Hugo Villafañe

**Affiliations:** 1 Department of Physiotherapy, Faculty of Sport Sciences, Universidad Europea de Madrid, 28670 Villaviciosa de Odón, Spain; joseluis.alonso@universidadeuropea.es (J.L.A.-P.); iker@centroethos.com (I.M.-P.); carlos.romero@universidadeuropea.es (C.R.-M.); vanesa.abuin@universidadeuropea.es (V.A.-P.); 2 Musculoskeletal Pain and Motor Control Research Group, Faculty of Sport Sciences, Universidad Europea de Madrid, 28670 Villaviciosa de Odón, Spain; 3 Department of Physical Medicine and Nursing, University of Zaragoza, 50009 Zaragoza, Spain; rlopezbu@unizar.es; 4 National Research Centre for the Working Environment, 2100 Copenhagen, Denmark; 5 Exercise Intervention for Health Research Group (EXINH-RG), Department of Physiotherapy, University of Valencia, 46100 Valencia, Spain; 6 Orthopaedics and Traumatology Unit, Clinica San Gaudenzio, Policlinico di Monza Group, Via Bottini 3, 28100 Novara, Italy; massimilianoleigheb@gmail.com

**Keywords:** musculoskeletal pain, fibromyalgia, chronic pain, vitamin D

## Abstract

Background/Objectives: Chronic pain impacts approximately 18% of the Spanish population, with low levels of vitamin D prevalent in over 80% of individuals over 65. Given vitamin D’s critical role in pain modulation, its deficiency may be significantly linked to chronic musculoskeletal pain, though existing research offers mixed results. Methods: This systematic review followed PRISMA guidelines, examining studies from PubMed, Cochrane, and PEDRO databases from 1990 onwards that investigated the relationship between vitamin D levels and chronic musculoskeletal pain. Results: A total of 30 studies met the inclusion criteria set by the NHLBI’s quality standards. The results are inconclusive regarding the direct relationship between vitamin D levels and chronic musculoskeletal pain due to evidence heterogeneity. However, there appears to be an inverse relationship between vitamin D levels and the intensity of pain. Conclusions: While the association between vitamin D levels and chronic musculoskeletal pain remains uncertain, the inverse correlation with pain intensity suggests a potential therapeutic role of vitamin D supplementation in pain management. Further research is needed to substantiate these findings and refine intervention strategies.

## 1. Introduction

Chronic pain affects approximately 18% of the population in Spain, with an estimated economic cost of 16,000 million euros, representing 25% of GDP (Gross Domestic Product). Chronic pain is defined as a stressful experience associated with existing or potential tissue damage, with sensory, cognitive, emotional, and social components [1]. This complexity makes the experience of pain a multifaceted phenomenon involving biochemical, neurological, and subjective factors, which increase susceptibility to the chronification of pain.

Among the various types of chronic pain, chronic musculoskeletal pain (CMP) is one of the most prevalent, significantly impacting individuals’ physical function and quality of life. CMP is characterized by pain originating in muscles, bones, joints, and connective tissues and is frequently associated with conditions such as osteoarthritis, rheumatoid arthritis, and fibromyalgia. In addition to biomechanical and inflammatory factors, nutritional deficiencies, particularly of vitamin D, have emerged as contributing factors to CMP [2,3].

Research into CMP has aimed to elucidate the mechanisms underlying pain chronification, including peripheral and central nociceptive sensitization, which involve prolonged activation and hypersensitivity of pain pathways [4,5,6]. Cognitive and emotional factors, such as catastrophic thinking and emotional dysregulation, also play critical roles in this process [7,8,9].

Vitamin D has garnered significant attention for its potential role in managing CMP. This secosteroid hormone is involved in calcium homeostasis and skeletal health, but it also modulates the immune system and inflammatory pathways. Vitamin D interacts with its receptor (VDR) in various immune and muscle cells, promoting the production of anti-inflammatory cytokines like IL-10, while reducing pro-inflammatory mediators such as TNFα and IL-1 [10]. In the context of CMP, vitamin D deficiency has been linked to increased pain perception, impaired muscle strength, and reduced muscle regeneration due to oxidative stress and mitochondrial dysfunction [11].

The recommended doses of vitamin D for musculoskeletal health vary depending on age, baseline serum levels, and geographic location. For individuals with deficiency (<20 ng/mL serum 25-hydroxyvitamin D), daily doses between 2000 and 4000 IU are generally suggested, while maintenance doses of 800–1200 IU per day are recommended for prevention in at-risk populations [12,13]. These doses are believed to be effective in improving muscle strength, reducing pain, and enhancing overall skeletal health, although optimal dosing for chronic musculoskeletal pain specifically requires further study.

In Spain, low vitamin D concentrations are highly prevalent, exceeding 80% in individuals over 65 years and 40% in younger populations. This widespread deficiency, combined with the mechanistic evidence linking vitamin D to pain modulation, highlights the need for further investigation into the relationship between vitamin D levels and CMP [14,15].

Previous studies have investigated this relationship with conflicting results. For example, one study in men aged 40–79 years found lower vitamin D levels in those with musculoskeletal pain [16]. However, systematic reviews have been inconclusive due to the heterogeneity of results.

The objective of this study is to investigate the relationship between serum vitamin D levels and CMP in adults. The study focuses on examining whether there is a direct correlation between low levels of vitamin D and the presence and intensity of CMP, using a systematic review of previous studies covering this topic. Additionally, it seeks to determine if vitamin D supplementation could play a therapeutic role in managing pain in these patients.

## 2. Materials and Methods

The systematic review was conducted following the PRISMA guidelines [17] and organized using the PICO framework. Searches were conducted in PubMed, Cochrane, and PEDro databases using the following keywords: “Musculoskeletal chronic pain”, “fibromyalgia”, “chronic widespread pain”, “vitamin D”, “vitamin D deficiency”, and “vitamin D levels”. Observational studies published between 1 January 1990 and 31 October 2022 were included.

### 2.1. Inclusion and Exclusion Criteria

Studies were included if they involved adults aged 18 years and older suffering from chronic musculoskeletal pain, with vitamin D levels assessed via blood tests. Exclusion criteria were studies involving participants under 18 years of age and studies evaluating pain other than chronic musculoskeletal pain.

### 2.2. Primary Outcomes

- Pain Intensity: Pain levels were quantified using validated tools such as the Visual Analogue Scale (VAS) and the Fibromyalgia Impact Questionnaire (FIQ). Specific thresholds for categorizing pain intensity (e.g., mild, moderate, severe) were established.
- Vitamin D Levels: Serum vitamin D concentrations were measured in ng/mL. Definitions for vitamin D deficiency (<20 ng/mL), insufficiency (20–30 ng/mL), and sufficiency (>30 ng/mL) were standardized according to the Endocrine Society guidelines.

### 2.3. Secondary Outcomes

- Quality of Life: Assessed using instruments such as the Short Form Health Survey (SF-36) and the EuroQol-5D (EQ-5D).
- Functional Ability: Measured using the Health Assessment Questionnaire (HAQ) and the Oswestry Disability Index (ODI).
- Biochemical Markers: Levels of parathyroid hormone (PTH), calcium, and phosphate were recorded to provide context on the metabolic impact of vitamin D deficiency.
- Sun Exposure and Dietary Intake: Evaluated using validated questionnaires and dietary intake records to assess other sources of vitamin D.
- Demographic and Baseline Characteristics: Data on age, gender, BMI, baseline pain levels, and comorbid conditions were collected to adjust for confounding variables.

### 2.4. Quality Assessment

The quality of the included studies was evaluated using the NIH Quality Assessment Tool for Observational Cohort and Cross-Sectional Studies. This tool consists of 14 criteria, with each item rated as “cannot determine”, “not applicable”, or “not reported”. Based on the overall scoring, studies were classified as “good” (11–14 points), “fair” (5–10 points), or “poor” (0–4 points). For case series, the NIH Quality Assessment Tool for Case Series Studies was applied.

### 2.5. Data Analysis

The data analysis involved grouping studies according to the presence or absence of a control group, the use of sun exposure questionnaires, and the assessment of pain levels in relation to vitamin D status. A qualitative analysis was conducted to explore the relationships between vitamin D levels and chronic musculoskeletal pain, as well as between vitamin D levels and pain intensity.

## 3. Results

### 3.1. Study Selection

A total of 30 observational studies investigating the relationship between vitamin D levels and chronic musculoskeletal pain in adults were reviewed following the PRISMA guideline. These studies were obtained by searching PubMed, Cochrane, and PEDro databases, covering the period from 1990 to 2022. The selected studies met the quality standards of the NHLBI guidelines.

### 3.2. Analysis and Synthesis of Results

The review focused on qualitatively examining the association between vitamin D levels and chronic musculoskeletal pain, differences between men and women, and the inverse relationship between vitamin D levels and pain intensity. It differentiated studies with control groups from those without, highlighting that those with control groups provide more reliable information. The review also considered the timing of blood sample collection due to seasonal variations in UVA radiation affecting vitamin D levels, reflecting this in the methodological quality of the studies, as shown in Table 1 [18].

#### 3.2.1. Vitamin D and Chronic Musculoskeletal Pain

In Table 1, the number of selected studies that investigated the relationship between vitamin D and chronic musculoskeletal pain with a control group is gathered, amounting to 12 studies. The total number of patients analyzed was 18,136, with at least 7468 men and at least 10,567 women; the gender distribution was not defined in the study by Block, S. R. [19] (he does not provide the data in the study). Out of these studies, De Giorgi et al. [11] found a relationship between chronic pain and low vitamin D levels (below 30 ng/mL) with a total of 18,035 participants, while one study found no relationship with a total of 101 participants.

By country, there are six studies in Turkey, three in Egypt, two each in Iran and Brazil, and one each in Portugal, Chile, Spain, Scotland, and Israel. In one study, Atherton et al. [22] analyzed comparative data between women and men, all born in the same year, finding a positive association between low vitamin D levels and chronic pain in women but not in men. This contrasts with the study by McBeth, J. et al., which found a positive association between low vitamin D levels and chronic pain in men of different ages. Both studies used the same criteria for chronic pain diagnosis (1990 ACR criteria) and did not report the pain scale used. The Ns were similar (3365 vs. 3369), but Atherton’s study was conducted in the UK, while McBeth’s was in several European countries. Atherton’s study provides data for blood collection between September 2002 and March 2004, while McBeth’s study does not [16]. In contrast, Table 2 clustered the selected studies that investigated the same topic without a control group, amounting to 18 studies with a total N of 2575 participants. The results showed differences between studies that found lower vitamin D levels in people with chronic pain and those that found no difference in vitamin D levels. Seven studies, with a total N of 1144 people, found lower vitamin D levels in people with chronic pain, of which 489 were women (lacking data from Heidari, B. et al., 2010, a mixed study with 496 participants). Eleven studies, with a total of 1431 participants (1428 women and 3 men), found no difference between the vitamin D levels of the two groups. These data gave more heterogeneous results than those without a control group, with many studies finding no relationship between vitamin D levels and chronic pain. The number of women analyzed was much higher than men, making gender comparisons difficult. Heidari’s study showed a greater positive association between vitamin D deficiency and pain in women, but the number of participants by gender was not provided for broader evaluation [30].

#### 3.2.2. Relationship Between Vitamin D and Chronic Musculoskeletal Pain Considering the Time of Sun Exposure

A recurring issue between possible low vitamin D levels and chronic pain was the possibility that low vitamin D levels were not a cause, but rather a consequence of the limitations that the pain itself could generate in the person, affecting their sun exposure [46,47].

Therefore, a relevant methodological factor to consider was the use of sun exposure questionnaires in the studies, which would allow us to assess a possible difference in exposure between groups. After reviewing all the studies in Table 2 and Table 3, we found four studies that did include sun exposure questionnaires.

In Table 3 we present the four studies that did take into account the sun exposure time of the participants. The first of the studies that did include the sun exposure questionnaire, Erkal, et al. [21] found that there was low sun exposure in the mixed group of participants analyzed. They concluded that there were low vitamin D levels in people with chronic pain and that there was low sun exposure. However, the absence of a control group made it impossible to compare whether there was actually lower sun exposure in the group tested compared to a control group without chronic pain.

In the second study, by Rezende Pena, C et al. 2010 [35], which included a sun exposure questionnaire and a control group, it was observed that the time of sun exposure was less in the patient group than in the control group, with no difference in the exposed body surface area. However, even with the difference in exposure, it was concluded that there was no difference in vitamin D levels between the two groups. It should be noted that there was a higher use of sunscreen in the control group, which may have affected vitamin D levels.

In the third study, which also included a control group and a sun exposure questionnaire, a calculation of the ultraviolet light index was conducted. The ultraviolet light index measures the exposure ratio in relation to the ability of the clothing worn to prevent exposure to solar radiation. The values obtained gave a non-significant statistical difference (*p* = 0.2) (the degree of exposure was between 0.63 and 0.03 in patients and between 0.62 and 0.06 in the control group). This study concluded that there was no difference in vitamin D levels between the patient group and the control group.

The fourth study that conducted a sun exposure questionnaire was conducted by Beserra, S.R. et al., 2020 [44]. The results regarding sun exposure showed that both the patient group and the control group had exposure of more than 30 min per day, or at least 3–4 h per week, reaching the minimum exposure in Brazil, a country with a high level of sunshine throughout the year. This study concluded that they found no difference in vitamin D levels between the two groups.

Once again, we observed a significant difference between the studies that had a control group and those without, in terms of the result and a possible association between low vitamin D levels and chronic musculoskeletal pain. We observe that in the studies where there was a control group, there is heterogeneity between one study that does observe a difference in sun exposure between groups and two that do not observe such a difference.

### 3.3. Study Quality Assessment

Study quality was assessed using the Quality Assessment Tool for Observational Cohort and Cross-Sectional Studies for cross-sectional studies and the Quality Assessment of Case-Control Studies for case-control studies. Most of the included studies met the minimum quality criteria, and several achieved a good quality assessment. However, heterogeneity in study designs and pain assessment methods limits the robustness of the conclusions, see Table 3.

**Table 3:** Analysis of the included studies.

Study	1	2	3	4	5	6	7	8	9	10	11	12	13	14	Total	Evaluación Calidad
McBeth, J. et al. (2010) [16]	Yes	Yes	NA	Yes	NR	Yes	Yes	Yes	Yes	NA	YES	NR	NA	Yes	9/11	GOOD
Block, S. R. (2004) [31]	Yes	NO	NA	Yes	NR	Yes	Yes	Yes	Yes	NA	YES	NR	NA	Yes	8/11	FAIR.
MacFarlane, G. J. et al. (2005) [38]	Yes	Yes	NA	Yes	NR	Yes	Yes	Yes	Yes	NA	YES	NR	NA	Yes	8/11	FAIR.
Erkal, M. Z. et al. (2006) [35]	Yes	Yes	NA	Yes	NR	Yes	Yes	Yes	Yes	NA	NO	NR	NA	Yes	8/11	FAIR
Atherton, K. et al. (2009) [32]	Yes	Yes	NA	Yes	NR	Yes	Yes	Yes	Yes	NA	NO	NR	NA	Yes	8/11	FAIR
Bhatty, S. A. et al. (2010) [39]	Yes	Yes	NA	Yes	NR	Yes	Yes	Yes	Yes	NA	NO	NR	NA	Yes	8/11	FAIR
Matthana, M. H. (2011) [40]	Yes	Yes	Yes	Yes	NR	Yes	Yes	Yes	Yes	Yes	Yes	NR	Yes	Yes	12/14	GOOD
Abokrysha, N. T. (2012) [41]	Yes	Yes	NA	Yes	NR	Yes	Yes	Yes	Yes	NA	Yes	NR	NA	Yes	9/11	GOOD
von Känel, R. et al. (2014) [42]	Yes	Yes	NA	Yes	NR	Yes	Yes	Yes	Yes	NA	Yes	NR	NA	Yes	9/11	GOOD
McCabe, P. S. et al. (2016) [43]	Yes	Yes	Yes	Yes	NR	Yes	Yes	Yes	Yes	Yes	Yes	NR	Yes	Yes	12/14	GOOD
Amin, O. A. et al. (2019) [44]	Yes	Yes	NA	Yes	NR	Yes	Yes	Yes	Yes	NA	Yes	NR	NA	Yes	9/11	GOOD
D’Souza, R. S. et al. (2020) [45]	Yes	Yes	Yes	Yes	NR	Yes	Yes	Yes	Yes	NO	Yes	NR	Yes	Yes	11/14	GOOD
Al-Allaf, A. W. et al. (2003) [19]	Yes	Yes	NA	Yes	NR	Yes	Yes	Yes	Yes	NA	Yes	NR	NA	Yes	9/11	GOOD
Tandeter, H. et al. (2009) [22]	Yes	Yes	NA	Yes	NR	Yes	Yes	Yes	Yes	NA	NO	NR	NA	Yes	8/11	FAIR
Ulusoy, H. et al. (2010) [46]	Yes	Yes	NA	Yes	NR	Yes	Yes	Yes	Yes	NA	Yes	NR	NA	Yes	9/11	GOOD
Heidari, B. et al. (2010) [33]	Yes	Yes	NA	Yes	NR	Yes	Yes	Yes	Yes	NA	Yes	NR	NA	Yes	9/11	GOOD
Maafi, A. A. et al. (2010) [47]	Yes	Yes	NA	Yes	NR	Yes	Yes	Yes	Yes	NA	Yes	NR	NA	Yes	9/11	GOOD
de Rezende Pena, C et al. (2010) [36]	Yes	Yes	NA	Yes	NR	Yes	Yes	Yes	Yes	NA	Yes	NR	NA	Yes	9/11	GOOD
Olama, S. M. et al. (2013) [21]	Yes	Yes	NA	Yes	NR	Yes	Yes	Yes	Yes	NA	Yes	NR	NA	Yes	9/11	GOOD
Okumus, M. et al. (2013) [20]	Yes	Yes	NA	Yes	NR	Yes	Yes	Yes	Yes	NA	Yes	NR	NA	Yes	9/11	GOOD
Mateos, F. et al. (2014) [23]	Yes	Yes	NA	Yes	NR	Yes	Yes	Yes	Yes	NA	NO	NR	NA	Yes	8/11	GOOD
Baygutalp, N. K. (2014) [24]	Yes	Yes	NA	Yes	NR	Yes	Yes	Yes	Yes	NA	Yes	NR	NA	Yes	9/11	GOOD
Elaziz Labeeb, A. A., & Al-Sharaki, D. R. (2015) [25]	Yes	Yes	NA	Yes	NR	Yes	Yes	Yes	Yes	NA	Yes	NR	NA	Yes	9/11	GOOD
Okyay, R. et al. (2016) [26]	Yes	Yes	NA	Yes	NR	Yes	Yes	Yes	Yes	NA	Yes	NR	NA	Yes	9/11	GOOD
Kasapoğlu Aksoy, M., et al. (2017) [27]	Yes	Yes	NA	Yes	NR	Yes	Yes	Yes	Yes	NA	Yes	NR	NA	Yes	9/11	GOOD
Lanas et al. (2017) [28]	NO	Yes	NA	Yes	NR	Yes	Yes	Yes	Yes	NA	Yes	NR	NA	Yes	8/11	FAIR
Beserra, S. R. et al. (2020) [37]	Yes	Yes	NA	Yes	NR	Yes	Yes	Yes	Yes	NA	Yes	NR	NA	Yes	9/11	GOOD
Akar, N. et al. (2020) [29]	Yes	Yes	NA	Yes	NR	Yes	Yes	Yes	Yes	NA	Yes	NR	NA	Yes	9/11	GOOD

NA: Not Applicable; NR: Not Reported.

#### Vitamin D and Pain Levels

Another research question was to check for a possible association between vitamin D levels and pain intensity. Across the studies reviewed, the total number of patients with pain was 9431, while the number in the control group was 733. Of the total number of patients, at least 4526 were women and at least 4099 were men (one study does not specify). Regarding pain scales, nine studies used the VAS, nine studies used the FIQ, and among these nine studies, seven used both scales for pain measurement. Additionally, one study used the Wong-Baker Faces Pain Scale, one used the WPI, one employed PEG sensitivity algometry, one used manikins, and one did not specify the pain scale used.

Out of the studies reviewed, 15 reported an association between lower vitamin D levels and increased pain scale scores. None of these studies specified a non-significant association. One study found no association between vitamin D levels and pain intensity on any scale.

Among the studies that reported an association between lower vitamin D levels and pain, two studies observed a higher incidence of this relationship in women compared to men, while the others did not specify gender-based differences.

## 4. Discussion

This systematic review addresses the relationship between serum vitamin D levels and CMP in adults. The evidence for a direct relationship between low vitamin D levels and the presence of CMP remains inconclusive. However, most studies that assessed pain intensity found an inverse relationship between vitamin D levels and pain intensity, suggesting that while vitamin D deficiency may not directly cause CMP, it could influence its severity.

In the results and discussion section, we noted the importance of potential confounding factors like sun exposure and activity rate. Sun exposure is a critical determinant of vitamin D synthesis, as ultraviolet B (UVB) radiation on the skin produces vitamin D. Variations in geographic location, season, clothing habits, and time spent outdoors could significantly influence serum vitamin D levels. For instance, individuals in higher latitudes or those with cultural practices that limit skin exposure to sunlight are more likely to have vitamin D deficiency. These differences may confound the observed association between vitamin D levels and CMP.

Physical activity rate is another significant confounder. Active individuals often experience improved overall health, which could influence both vitamin D status (due to increased sun exposure) and pain intensity (due to better musculoskeletal health). Importantly, physical activity itself has demonstrated analgesic effects in CMP conditions, further complicating the isolation of vitamin D’s role in this context [48]. Studies that do not account for these factors risk overestimating or underestimating the relationship between vitamin D and pain outcomes [49].

Future research should include detailed and standardized assessments of sun exposure, accounting for factors such as seasonal variations, sunscreen use, and clothing. Additionally, quantifying physical activity using reliable metrics will provide a clearer understanding of its role as a confounder.

One of the main strengths of this review is its systematic approach, which included a wide range of observational studies, providing a comprehensive view of the topic. Quality assessment tools further strengthen the reliability of the results.

However, methodological variability among the included studies limits direct comparisons and the synthesis of findings. Many studies lacked control groups, which weakens the ability to establish causal relationships. Additionally, the absence of specific data on sun exposure and activity levels further complicates result interpretation. These limitations highlight gaps in the current literature that must be addressed to draw more robust conclusions.

The results of this review are consistent with some previous reviews that found mixed evidence on the relationship between vitamin D levels and chronic pain. For example, previous reviews have found both evidence of a positive relationship and studies reporting no significant association. The review by Martins et al. [50] and Ali et al. [51] also concluded that the evidence is inconclusive, while the review by Ellis et al. [52] suggests a possible positive relationship between vitamin D deficiency and pain in fibromyalgia patients.

### 4.1. Clinical Relevance and Future Perspectives

The inverse relationship between vitamin D levels and pain intensity found in most studies suggests that correcting vitamin D deficiency could be a useful intervention to reduce pain intensity in patients with CMP [53,54,55,56]. This is particularly relevant given the high prevalence of vitamin D deficiency, especially among older adults.

For clinicians, assessing and correcting vitamin D levels in patients with CMP could provide a simple, safe, and cost-effective strategy to improve outcomes. However, it is crucial to consider confounding factors like sun exposure and physical activity when interpreting vitamin D levels. This underscores the need for a comprehensive approach to evaluate the potential benefits of vitamin D supplementation.

Future studies should prioritize longitudinal designs with control groups and standardized methods for measuring both serum vitamin D and pain intensity. Detailed assessments of sun exposure and physical activity are crucial for reducing confounding factors and clarifying the relationship between vitamin D and CMP. Long-term studies assessing the impact of vitamin D supplementation on pain outcomes will provide essential insights to inform clinical practice.

### 4.2. Limitations

This review has notable limitations, including potential publication bias, as studies with positive results are more likely to be published. Additionally, only studies published in English were included, potentially excluding relevant findings in other languages. The heterogeneity of study designs and methods precluded a detailed quantitative analysis, limiting the precision of our conclusions.

## 5. Conclusions

In conclusion, while the relationship between low vitamin D levels and CMP is not fully established, the evidence suggests that vitamin D may influence pain intensity. This review highlights the need for future research to address key confounding factors, such as sun exposure and activity rate, to enhance our understanding of this relationship. Considering vitamin D as part of CMP management strategies could provide significant benefits, particularly for populations at risk of deficiency.

## Figures and Tables

**Table 1:** Characteristics of the included studies without control group.

Authors	Design	N (M/F)	Diagnostic Criteria for Pain or Fibromyalgia (FM)	Pain Assessment	Vitamin D Cut-Off Levels	Is There a Relationship Between Vitamin D Levels and Chronic Pain?
Block, S. R. (2004) [19]	Case-control	101 (Not specified)	ACR 1990 Criteria	Not specified	2011 Endocrine Society (ES) Guidelines	No link between low levels of Vitamin D and FM
MacFarlane, G. J. et al. (2005) [20]	Cross-sectional	3135 (All female)	ACR 1990 Criteria	Signaling pain on mannequins	Not specified	Yes, Vitamin D deficits should be considered in people with chronic widespread pain.
Erkal, M. Z. et al. (2006) [21]	Cross-sectional	994 (M: 589, F: 405)	Not specified	Not specified	Deficiency: <25 nmol/L; Insufficiency: <50 nmol/L	Yes, link between low Vitamin D levels and pain.
Atherton, K. et al. (2009) [22]	Cross-sectional	6824 (M: 3365, F: 3459)	ACR 1990 Criteria	Not specified	Not specified	Vitamin D levels associated with chronic widespread pain in women but not in men.
Bhatty, S. A. et al. (2010) [23]	Cross-sectional	40 (All female)	ACR 1990 Criteria	Not specified	2011 Endocrine Society (ES) Guidelines	Yes, Vitamin D deficiency is common in FM.
McBeth, J. et al. (2010) [16]	Cross-sectional	3369 (All male)	ACR 1990 Criteria	Not specified	Low: <15 ng/mL; Normal: >15 ng/mL	Yes, association between low Vitamin D levels and chronic pain.
Matthana, M. H. (2011) [24]	Cohort study	100 (All female)	ACR 1990 Criteria	Fibromyalgia Impact Questionnaire (FIQ)	Not specified	Yes, Vitamin D deficiency has to be considered in FM.
Abokrysha, N. T. (2012) [25]	Cross-sectional	30 (All female)	ACR 2010 Criteria	Widespread Pain Index (WPI)	2011 Endocrine Society (ES) Guidelines	Yes, significant inverse relationship between Vitamin D levels and chronic disseminated pain.
von Känel, R. et al. (2014) [26]	Cross-sectional	174 (M: 81, F: 93)	ACR 2010 Criteria	PEG sensitivity algometry	2011 Endocrine Society (ES) Guidelines	Yes, high prevalence of hypovitaminosis in chronic pain patients.
McCabe, P. S. et al. (2016) [27]	Cohort study	2736 (All female)	ACR 1990 Criteria	Widespread Pain Index (WPI)	2011 Endocrine Society (ES) Guidelines	Yes, low Vitamin D levels are linked to the development of chronic widespread pain.
Amin, O. A. et al. (2019) [28]	Cross-sectional	40 (M: 8, F: 32)	ACR 2010 Criteria	Fibromyalgia Impact Questionnaire (FIQ)	2011 Endocrine Society (ES) Guidelines	Yes, although not significant.
D’Souza, R. S. et al. (2020) [29]	Cohort study	593 (M: 56, F: 537)	ACR 1990 Criteria	Fibromyalgia Impact Questionnaire (FIQ)	Low: ≤25 ng/mL; Adequate: >25 ng/mL	Yes, common hypovitaminosis in FM patients.

**Table 2:** Characteristics of the included studies with control group.

Authors	Study Design	N (M/F)	Diagnostic Criteria for Pain or Fibromyalgia (FM)	Pain Assessment	Vitamin D Cut-Off Levels	Is There a Relationship Between Vitamin D Levels and Chronic Pain?
Al-Allaf, A. W. et al. (2003) [31]	Cross-sectional	51 (42 female)	ACR 1990 Criteria	FIQ	Deficiency: <20 ng/mL; Normal: ≥20 ng/mL	Yes
Tandeter, H. et al. (2009) [32]	Cross-sectional	68 (82 female)	ACR 1990 Criteria	Not specified	Deficiency: <15 ng/mL; Insufficiency: <20 ng/mL	No
Ulusoy, H. et al. (2010) [33]	Cross-sectional	15 (15 female)	ACR 2010 Criteria	FIQ, VAS	Deficiency: <20 ng/mL; Severe deficiency: ≤8 ng/mL	No
Heidari, B. et al. (2010) [30]	Cross-sectional	276 (220 female)	Non-specific pain for at least 2 months	Wong-Baker Face scale	Deficiency: <20 ng/mL	Yes, positive association between Vitamin D deficiency and pain, especially in women in certain areas of the body
Maafi, A. A. et al. (2010) [34]	Cross-sectional	74 (68 female)	ACR 2010 Criteria	FIQ, Illness invalidation inventory	2011 Endocrine Society (ES) Guidelines	No
de Rezende Pena, C et al. (2010) [35]	Cross-sectional	87 (92 female)	ACR 1990 Criteria	VAS	Not specified	No
Olama, S. M. et al. (2013) [36]	Case-control	50 (50 female)	ACR 1990 Criteria	FIQ, VAS	Deficiency: <20 ng/mL; Severe deficiency: <8 ng/mL	Yes, more patients with lower Vitamin D levels in FM
Okumus, M. et al. (2013) [37]	Cross-sectional	40 (40 female)	Not specified	FIQ, VAS	Deficiency: <37.5 nmol/L	No. Similar levels in both groups but differences in pain intensity
Mateos, F. et al. (2014) [38]	Cross-sectional	205 (205 female)	Not specified	Not specified	Not specified	No
Baygutalp, N. K. (2014) [39]	Cross-sectional	19 (24 female)	ACR 1990 Criteria	FIQ, VAS	Not specified	Yes, low Vitamin D levels in patients with FM
Elaziz Labeeb, A. A., & Al-Sharaki, D. R. (2015) [40]	Cross-sectional	53 (50 female)	ACR 1990 Criteria	FIQ, VAS	Deficiency: <30 ng/mL; Normal: ≥30 ng/mL	Yes
Okyay, R. et al. (2016) [41]	Case-control	79 (80 female)	ACR 1990 criteria	FIQ, VAS	Deficiency: <20 ng/mL	Yes, lower Vitamin D levels in FM group than the control group
Kasapoğlu Aksoy, M., Altan, L., & Ökmen Metin, B. (2017) [42]	Cross-sectional	70 (65 female)	ACR 1990 criteria	FIQ, VAS	Cut-off above and below 30 ng/mL	No differences in Vitamin D levels between groups, but differences in pain intensity levels
Lanas et al. (2017) [43]	Case-control	39 (39 female)	ACR 2010 criteria	VAS	2011 Endocrine Society (ES) Guidelines	No
Beserra, S. R. et al. (2020) [44]	Cross-sectional	40 (43 female, 3 male)	ACR 2010 criteria	FIQ, VAS	2011 Endocrine Society (ES) Guidelines	No differences in Vitamin D levels between groups
Akar, N. et al. (2020) [45]	Cross-sectional	40 (40 female)	ACR 2010 criteria	VAS	Deficiency: <25 ng/mL; Normal: ≥25 ng/mL	Yes, higher hypovitaminosis frequency in FM group
